# A Multiscale Molecular Dynamic Analysis Reveals the Effect of Sialylation on EGFR Clustering in a CRISPR/Cas9-Derived Model

**DOI:** 10.3390/ijms23158754

**Published:** 2022-08-06

**Authors:** Shwee Khuan Leong, Jye-Chian Hsiao, Jiun-Jie Shie

**Affiliations:** 1 Institute of Chemistry, Academia Sinica, Taipei 11529, Taiwan; 2 Taiwan International Graduate Program (TIGP), Sustainable Chemical Science & Technology (SCST), Academia Sinica, Taipei 11529, Taiwan; 3 Department of Applied Chemistry, National Yang Ming Chiao Tung University (NYCU), Hsinchu 30050, Taiwan

**Keywords:** CRISPR/Cas9, sialylation, EGFR clustering, dynamic molecular study, number and brightness (N&B) analysis, raster image correlation spectroscopy (RICS)

## Abstract

Bacterial and viral pathogens can modulate the glycosylation of key host proteins to facilitate pathogenesis by using various glycosidases, particularly sialidases. Epidermal growth factor receptor (EGFR) signaling is activated by ligand-induced receptor dimerization and oligomerization. Ligand binding induces conformational changes in EGFR, leading to clusters and aggregation. However, information on the relevance of EGFR clustering in the pattern of glycosylation during bacterial and viral invasion remains unclear. In this study, (1) we established CRISPR/Cas9-mediated GFP knock-in (EGFP-KI) HeLa cells expressing fluorescently tagged EGFR at close to endogenous levels to study EGF-induced EGFR clustering and molecular dynamics; (2) We studied the effect of sialylation on EGF-induced EGFR clustering and localization in live cells using a high content analysis platform and raster image correlation spectroscopy (RICS) coupled with a number and brightness (N&B) analysis; (3) Our data reveal that the removal of cell surface sialic acids by sialidase treatment significantly decreases EGF receptor clustering with reduced fluorescence intensity, number, and area of EGFR-GFP clusters per cell upon EGF stimulation. Sialylation appears to mediate EGF-induced EGFR clustering as demonstrated by the change of EGFR-GFP clusters in the diffusion coefficient and molecular brightness, providing new insights into the role of sialylation in EGF-induced EGFR activation; and (4) We envision that the combination of CRISPR/Cas9-mediated fluorescent tagging of endogenous proteins and fluorescence imaging techniques can be the method of choice for studying the molecular dynamics and interactions of proteins in live cells.

## 1. Introduction

The receptor tyrosine kinase (RTK) family represents an important class of therapeutic targets in cancer therapy because their aberrant signaling has been linked to the formation of solid-state tumors and metastases [1]. Many studies have been conducted to elucidate the underlying mechanism by which RTK is activated, particularly focusing on receptor signaling, trafficking, internalization and downstream regulation [2]. Epidermal growth factor receptor (EGFR), a member of the RTK family, is highly dynamic in receptor assembly, and its activation and subsequent signal transduction are responsible for multiple cellular processes, such as proliferation, differentiation, apoptosis and angiogenesis [3]. Ligand-induced EGFR activation relies on protein conformational changes through ligand binding that triggers receptor clustering, such as the formation of dimers or oligomers [4,5].

Sialic acid residues (sialosides) on the cell surface can mediate influenza virus binding and entry into the cell [6] as they are responsible for intermolecular interactions, particularly in host recognition during bacterial and viral infection [7]. EGFRs are heavily glycosylated transmembrane proteins with multiple sialic acid residues typically located at the terminal end of glycan structures [8]. Notably, EGFR has been demonstrated to be a co-receptor for viral infection [9,10] and to transduce signals relevant to virus entry [11,12]. Influenza virus A binding was found to cluster lipid raft microdomains where EGFR was also highly co-localized, similar to which occurred in EGF stimulation [11], implying that EGFR also clusters to help virus entry. On the other hand, various viral and bacterial sialidases can cleave α-2,3, α-2,6 or α-2,8 sialosides on membrane-associated glycoconjugates [13] and could affect EGFR signaling during their infection [11,12]. Indeed, changing the glycan form has been shown to exert a substantial effect on EGFR functions [14,15], implying that terminal sialosides play a role in EGF-initiated EGFR activation and signaling [16,17,18,19,20]. Therefore, desialylation of surface glycans on host cells by pathogenic sialidases might also affect EGFR signaling. However, the effect of desialylation on EGFR dynamics regarding clustering and intracellular trafficking in live cells has rarely been reported. In this study, we specifically aimed to study the intracellular EGFR dynamics after global desialylation by exogenous sialidase, which can mimic the action of pathogenic sialidases during infection.

Visualizing protein dynamics is essential for understanding the molecular mechanism behind ligand-driven receptor activation and protein–protein interactions in live cells. Current live cell imaging studies generally rely on the overexpression of a target protein fused to a fluorescent protein. However, an overexpressed protein may overwhelm the endogenous protein stoichiometry, leading to cellular defects and artifacts such as protein mislocalization and artificial protein–protein interactions [21]. CRISPR/Cas9-guided genome editing is an emerging biotechnology that has been rapidly expanding in oncology and cancer therapies [22]. The CRISPR/Cas9 knock-in (KI) editing can enable site-directed insertion of a tagged protein of interest without disturbing the endogenous transcription machinery and hence the gene expression level [23]. To overcome the inherent limitations of protein overexpression, we applied CRISPR/Cas9 genome editing to establish a HeLa cell line (EGFP-KI) that expresses green fluorescent protein (GFP)-tagged EGFR (EGFR-GFP) at close to endogenous levels.

Fluorescence fluctuation spectroscopy (FFS) techniques such as raster image correlation spectroscopy (RICS) are useful to monitor and characterize real-time protein clustering, which relies on the statistical analysis of the fluctuation of a fluorescent signal to provide information about molecular diffusion. RICS is a powerful method that provides both spatial and temporal information, and the molecular interactions can be calculated with diffusion coefficients from a series of raster images using an autocorrelation function [24]. The molecular brightness is another characteristic variable that correlates highly with the state of a fluorescently-tagged protein cluster. Various approaches are available to derive molecular brightness, including single-particle tracking, fluorescence correlation spectroscopy, photon-counting histogram, and number and brightness (N&B) analysis [25]. N&B analysis uses the pixel-by-pixel fluorescence intensity fluctuation, which can be acquired simultaneously by RICS to derive the molecular brightness of a fluorophore and the corresponding brightness map (B map). In the present work, we studied the dynamics of EGF-induced EGFR clustering in EGFP-KI cells by RICS/N&B analysis in combination with confocal fluorescence imaging and high-content analysis. The results show that at the endogenous level, EGF-induced EGFR internalization and clustering were suppressed by the removal of cell surface sialic acid residuals by sialidase, accompanied by decreased molecular brightness and increased diffusion coefficients compared to those in EGF-treated only cells, suggesting that the sialic acid modification of cell surface glycan plays a substantial role in EGF-induced EGFR clustering.

## 2. Results

### 2.1. CRISPR/Cas9 Gene Editing for Constructing EGFP-KI HeLa Cells

**Figure 1:**
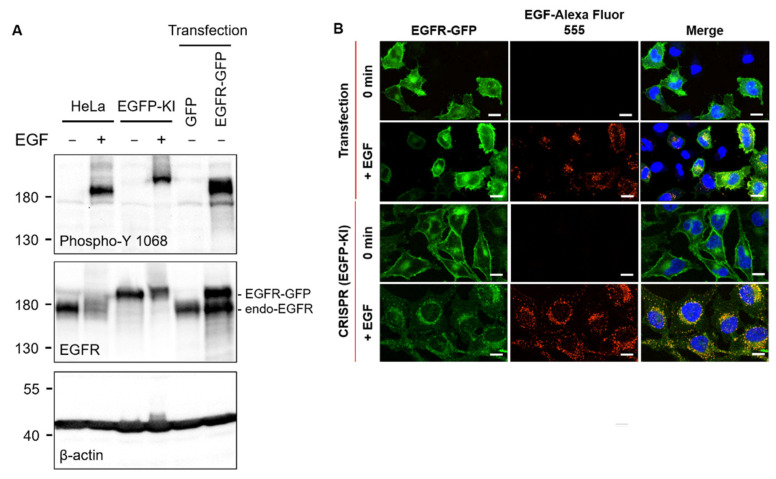
Comparison of EGFR-GFP in transfected (overexpression) and CRISPR/Cas9-mediated EGFP-KI HeLa cells. (**A**) Autophosphorylation levels at Tyr1068 of EGFR-GFP in transfected and EGFP-KI cells. Wild-type HeLa cells were used as a negative control; (**B**) Confocal fluorescence microscopy assessment of EGFR-GFP distribution and co-localization with EGF in live cells treated with EGF-Alexa Fluor 555 (15 min, 37 °C). Scale bar, 20 µm.

Among the generally used fluorescent tagging approaches, transient transfection is typically used to overexpress the fusion protein. However, protein overexpression is prone to aberrations such as protein mislocalization, malfunction, aggregation and violations of a balanced gene dosage [21]. Plasmid-driven transfection is known to promote the formation of undesired artifacts compared to the CRISPR/Cas9 approach [21,23]. We used CRISPR/Cas9-guided genome editing to generate a HeLa cell line with the knock-in expression of GFP-tagged EGFRs (EGFP-KI) at close to an endogenous level without overexpression-associated artifacts, allowing us to investigate molecular dynamics in a nearly native state. Figure 1 EGFP-KI and transiently transfected (plasmid-based) HeLa cells were compared by Western blot analysis and live cell imaging using fluorescence confocal microscopy. The Western blot results show a contrasting expression profile of the Tyr1068 autophosphorylation levels on EGFR and the corresponding EGFR protein levels between EGFP-KI and EGFR-GFP-transfected HeLa cells (Figure 1A). Notably, the protein expression level of EGFR-GFP in EGFP-KI cells was close to that of wild-type EGFR. In contrast to those of wild-type and EGFP-KI cells in which phosphorylation was barely detected without EGF treatment, EGFR phosphorylation at Tyr1068 in EGFR-GFP-overexpressing HeLa cells was constitutively expressed at high levels even without EGF, indicating aberrant activation of EGFR in overexpressed cells.

In our live cell experiments, a concentration of 100 ng/mL EGF as a dose within the tolerated range [26] was used to activate EGFR. We tested whether EGF treatment caused ligand-induced receptor internalization in live cells (Figure 1B). The distribution of fluorescence intensity of overexpressed EGFR-GFP across transfected cells was nonhomogeneous and did not coincide with EGF after treatment. In contrast, EGFR-GFP in EGFP-KI cells showed a relatively consistent distribution throughout each cell and was highly co-localized with EGF when internalized. These results indicate that EGFR fused with GFP at the C-terminus remains the endocytic behaviors of endogenous receptors and hence is an appropriate live cell platform to study EGFR internalization. Additionally, the inclusion of 26 kDa GFP does not alter EGF-induced conformational rearrangements of receptor molecules [27].

**Figure 2:**
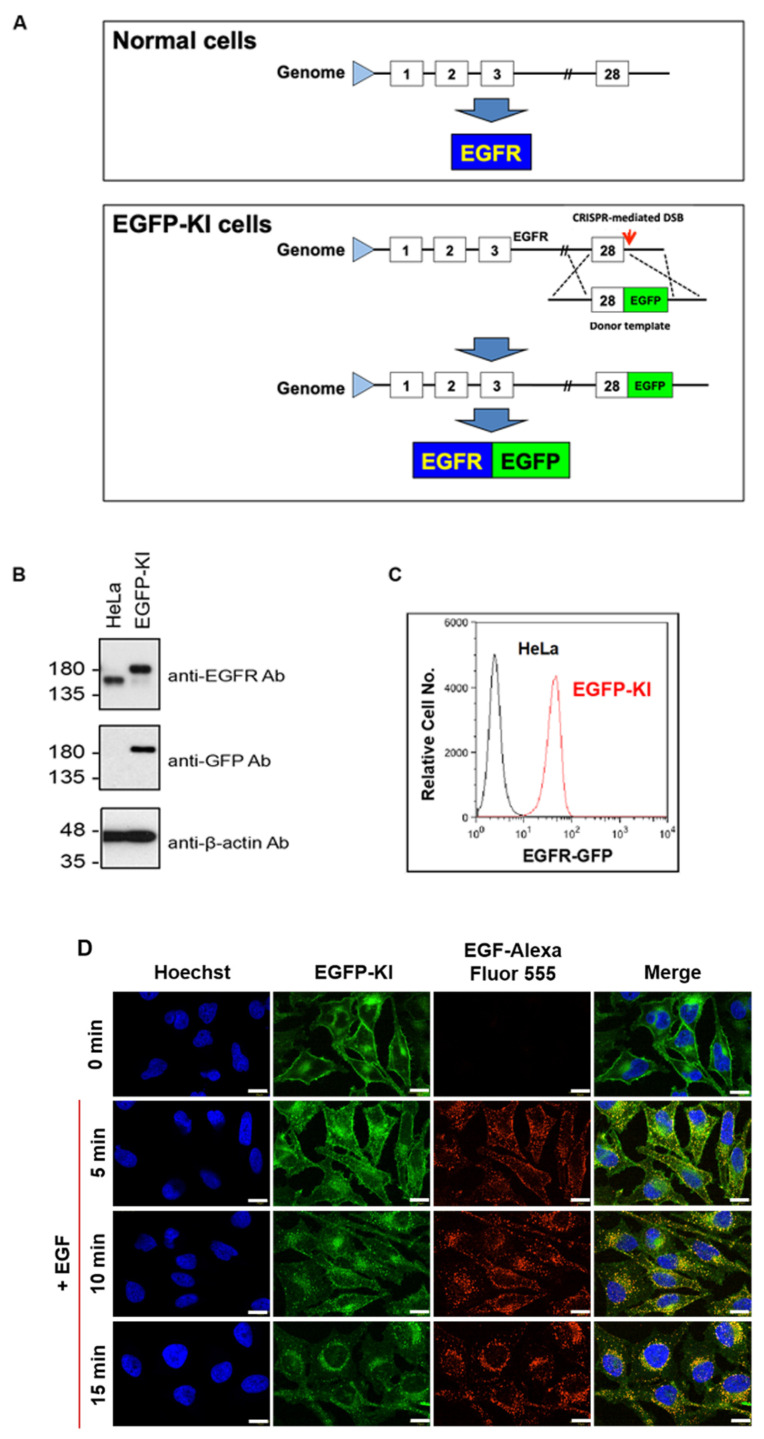
Construction of CRISPR/Cas9-mediated EGFP-KI HeLa cells. (**A**) Schematic representation of the EGFR-GFP construct with a knock-in EGFP segment at the C-terminus. Boxes represent EGFR exons and the number indicates the exon numbering; (**B**) Western blot analysis of EGFR-GFP in EGFP-KI cells against EGFR and GFP; (**C**) Flow cytometry analysis of the EGFR-GFP fluorescence intensity; (**D**) Time-course fluorescence images of EGFP-KI cells upon treatment with 100 ng/mL EGF at 37 °C for up to 15 min. Scale bar, 20 µm.

As illustrated in Figure 2A, the CRISPR/Cas9 editing of the EGFR gene locus is mediated by double-strand break (DSB) repair with homologous recombination. The donor plasmid carries homologous arms (HA-L and HA-R) of the CRISPR/Cas9 cut site flanking a fluorescent reporter gene (GFP). In the presence of a donor plasmid, homologous recombination uses the donor as a template to repair a DSB, achieving targeted integration. The expression of fluorescently tagged GFP fused to EGFR was further characterized by Western blot analysis, as shown in Figure 2B. The Western blot analysis of EGFP-KI lysates demonstrated an up-shifted band at approximately 180 kDa using EGFR antibodies in combination with GFP compared to wild-type HeLa cells, indicating stable expression of EGFR-GFP at close to the endogenous level. CRISPR-derived EGFP-KI cells were analyzed by flow cytometry to confirm the successful tagging of GFP fluorescence (Figure 2C). We then tested to ensure the molecular interaction and recognition of EGFR were not compromised by the tagged GFP by assessing treatment with the fluorescently labeled EGF ligand shown in Figure 2D. The fluorescence signal of EGFR was found to highly co-localize with EGF irrespective of treatment time. Additionally, EGF induced significant EGFR internalization, particularly after 10-min of treatment. This observation indicates that engineered EGFR-GFP on the surface retains the ability to recognize EGF and form receptor clusters that induce clathrin-dependent or clathrin-independent endocytosis for degradation and recycling [28]. The complex structural organization of EGFR distributed on the cell membrane is fundamental for physiological roles involving ligand- and kinase-dependent activation. We successfully engineered EGFR by CRISPR EGFP-KI HeLa cells to provide stable expression of EGFR-GFP for further RICS and N&B measurements and analysis.

### 2.2. Metabolic Labeling of Sialic Acid by Strain-Promoted Azide-Alkyne Cycloaddition (SPAAC) to Confirm EGFR Desialylation by Sialidase

**Figure 3:**
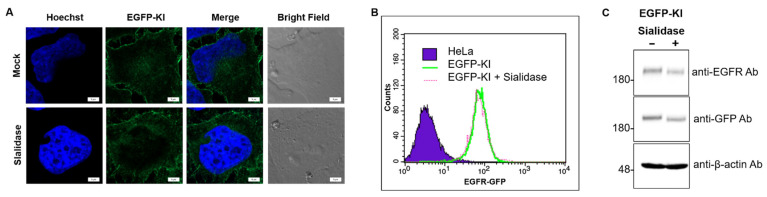
Effects of sialidase on EGFR-GFP in EGFP-KI cells. (**A**) Confocal fluorescence images of EGFP-KI cells with and without prior treatment with sialidase (3 U/mL) for 1 h at 37 °C. Sialidase did not affect the distribution or fluorescent signal of EGFR-GFP. Scale bar, 5 µm; (**B**) Flow cytometry analysis of EGFP-KI cells with and without prior sialidase treatment; (**C**) Western blot analysis of EGFR-GFP levels in EGFP-KI cells with and without prior sialidase treatment.

Sialylation is an important type of glycosylation as sialylated glycoconjugates on the cell surface play a vital role in cellular recognition, cell signaling and cell adhesion [29]. Several biochemical studies have reported that sialylation of EGFR affects the activation and subsequent signaling [16,17,18,19,20]. However, the effect of sialylation on EGF-induced EGFR clustering and localization in live cells still remains elusive. An enzymatic approach using sialidases is often employed to remove the terminal sialic acid from the glycan chain in a regioselective manner. We initially examined whether sialidase-mediated desialylation at α-2,3, α-2,6 and α-2,8 linkages altered the distribution of surface EGFRs in EGFP-KI cells (Figure 3). Live cell fluorescence imaging (Figure 3A) and flow cytometry analysis (Figure 3B) indicated no obvious change in EGFR-GFP fluorescence intensity and distribution around the cell after sialidase treatment. Sialidase had little effect on the protein expression level, as demonstrated by the Western blot analysis in Figure 3C.

**Figure 4:**
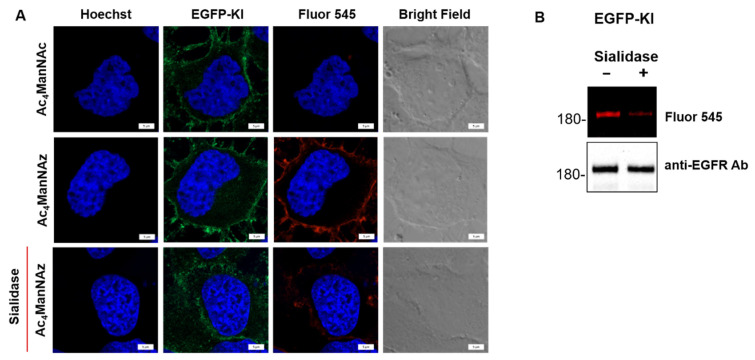
Confirmation of EGFR-GFP desialylation by sialidase in EGFP-KI cells using SPAAC. (**A**) Fluorescence images of azido sialic acids (SiaNAz) labeling with Fluor 545 through SPAAC in EGFP-KI cells (pretreated with Ac_4_ManNAz for 3 days) and the effect of sialidase (3 U/mL) on the labeling. Scale bar, 5 µm; (**B**) In-gel fluorescence analysis of Fluor 545-labeled EGFR-GFP immunoprecipitation-enriched EGFP-KI cell lysates with and without prior sialidase treatment.

We further examined whether the sialic acids on EGFR-GFP were removed by the enzymatic action of sialidase in live EGFP-KI cells. For this purpose, EGFP-KI cells were incubated with azido-sugar (Ac_4_ManNAz), which can be deacetylated by cytosolic esterase, and through the endogenous biosynthesis pathway, metabolically converted into *N*-azidoacetyl sialoside (SiaNAz) present on cell surface glycans [30]. With an exogenous cyclooctyne-bearing fluorescent probe (Fluor 545), the azido group of SiaNAz can be fluorescently labeled by the SPAAC reaction [31]. We compared the fluorescent labeling of cell surface SiaNAz in EGFP-KI cells with and without sialidase treatment in live cell imaging and using in-gel fluorescence analysis. Ac_4_ManNAc, which can transform into sialic acid, was used for the control experiment. In live cell imaging, the red fluorescent signal derived from Fluor 545 labeling of SiaNAz-bearing glycoconjugates was evident on the plasma membrane in Ac_4_ManNAz-treated cells. Furthermore, it was highly co-localized with GFP-tagged EGFRs (green fluorescence). In contrast, sialidase treatment significantly reduced the fluorescent signal (Figure 4A and Figure S2 in the Supplementary Information). In-gel fluorescence analysis further indicated that the Fluor 545 signal of EGFR-GFP (enriched from EGFP-KI cell lysates using immunoaffinity beads) had a reduced fluorescent signal in the sialidase-treated group (Figure 4B). Altogether, these results confirmed that sialidase could remove the α-2,3, α-2,6 and α-2,8 sialosides from EGFR-GFP on the membrane of EGFP-KI cells.

### 2.3. Effect of Sialidase on EGF-Induced Internalization of EGFR Clusters

**Figure 5:**
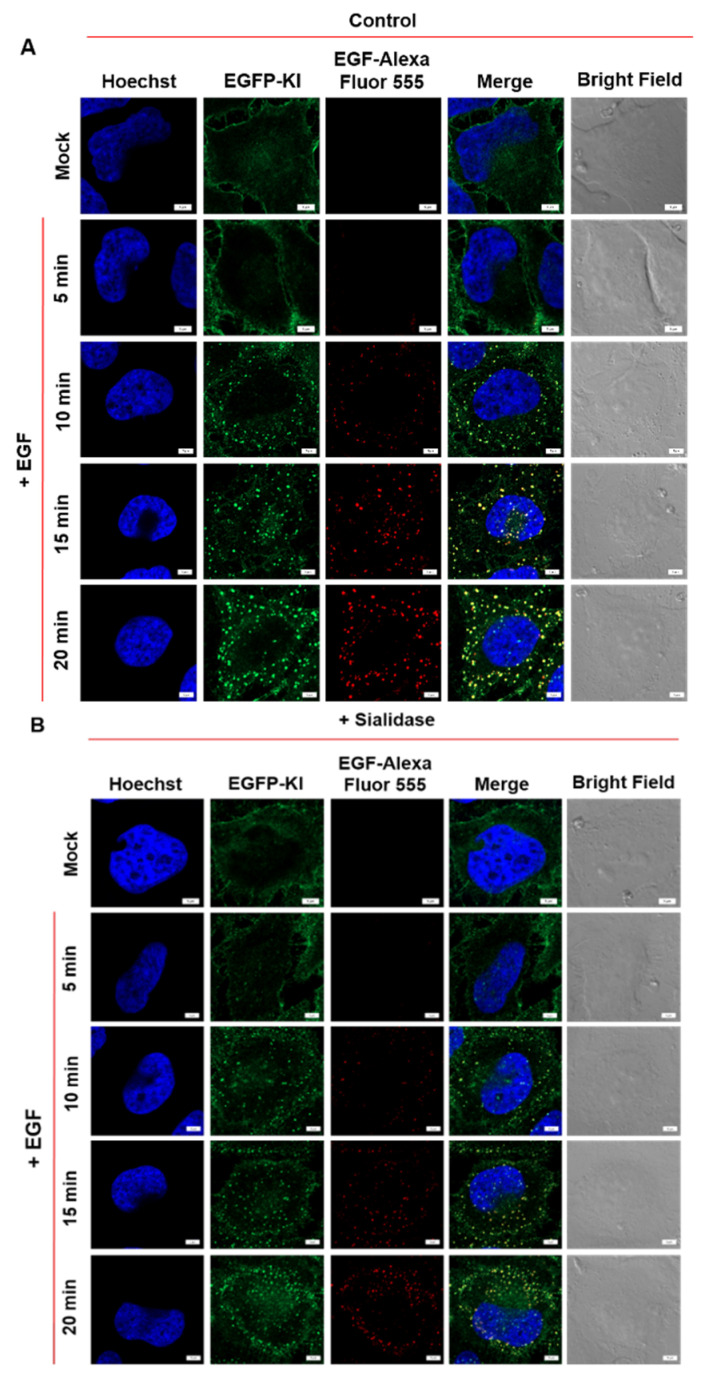

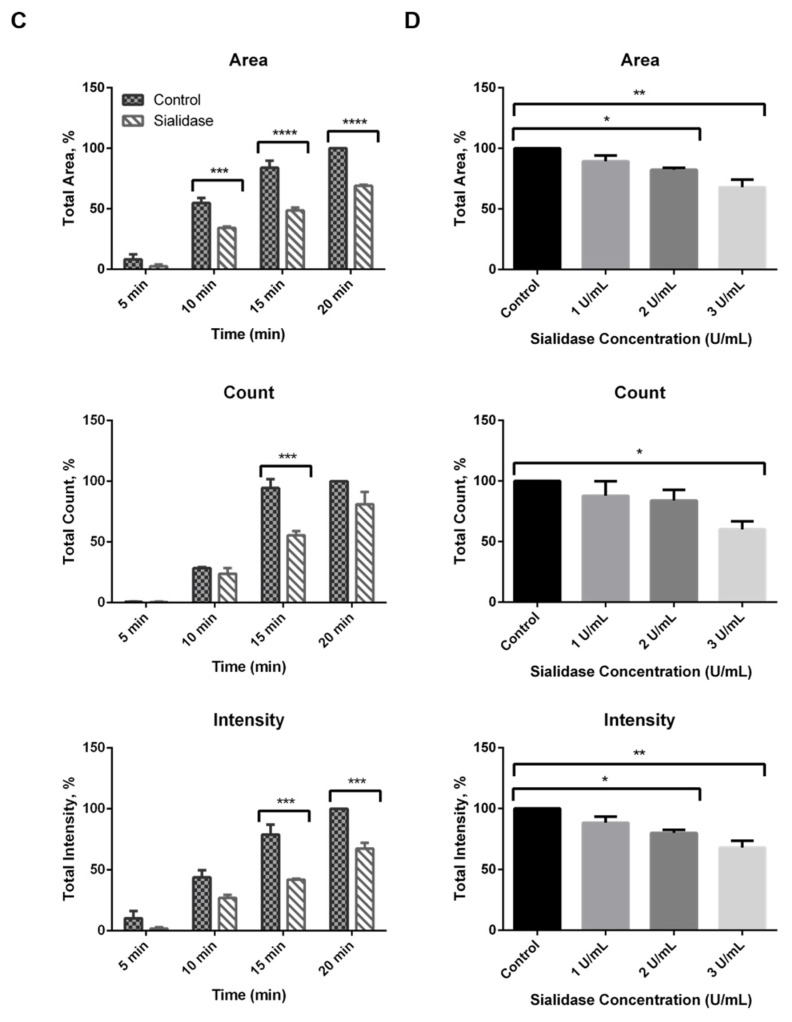
Effects of desialylation on EGF-induced internalization of EGFR-GFP clusters in EGFP-KI cells. (**A**) Time-lapse fluorescence images of EGFP-KI cells treated with 100 ng/mL EGF-Alexa Fluor 555 for 5–20 min at 37 °C; (**B**) EGFP-KI cells pretreated with sialidase (3 U/mL for 1 h) followed by 100 ng/mL EGF-Alexa Fluor 555. Scale bar, 5 µm; (**C**) Time-dependent high content analysis of normalized total fluorescence intensity, count and area of EGFR clusters per cell; values are reported as the means ± SEM of three independent experiments; (**D**) Dose-dependency of EGF-induced EGFR-GFP internalization on sialidase; values are reported as the means ± SEM of three independent experiments. Each value was normalized to the highest value for comparison purposes. *p* values were calculated using two-way ANOVA with * *p* ≤ 0.05, ** *p* ≤ 0.01 *** *p* ≤ 0.001, and **** *p* ≤ 0.0001 considered significant.

We evaluated whether EGFR-induced EGFR-GFP internalization would be affected in desialylated EGFP-KI cells by employing confocal fluorescence imaging in combination with high content analysis. (Figure 5 and Figure S3 in the SI). EGF-induced internalization and clustering were assessed by time-lapse fluorescence imaging in live cells at intervals of 5 min (Figure 5A and Figure S3A in the SI). Initially, the green fluorescence of EGFR-GFP was mainly located on the plasma membrane, and following EGF treatment, it became clusters that mainly accumulated inside the cell and co-localized with EGF-Alexa Fluor 555, which implies EGF-induced receptor clustering and internalization. The cluster density (the number of clusters per area) and fluorescence intensity of the EGFR-EGF complex were increased in a time-dependent manner. Compared to control cells, the desialylated cells with sialidase formed significantly smaller EGFR clusters (Figure 5B and Figure S3B in the SI). For quantification analysis, we employed a high-content imaging and analysis platform to compare the internalized EGFR cluster signals with and without sialidase treatment. We evaluated the internalized EGFR-GFP clusters with respect to the fluorescence intensity, count and size of each living cell after EGF stimulation. The quantitative results showed significant time-dependent increases in the normalized total number and size, as well as the fluorescence intensity of EGFR clusters, following EGF stimulation. Interestingly, EGFR clustering was affected in sialidase-treated cells with reduced clusters in total area, count, and fluorescence intensity after EGF treatment (Figure 5C). Furthermore, EGFR-KI cells were treated with different doses of sialidase (1, 2 and 3 U/mL) for 1 h, followed by EGF stimulation for 15 min. A dose-dependent decrease in EGFR-GFP clustering on sialidase was demonstrated, as shown in Figure 5D. Altogether, these observations indicate that the sialic acid modification on the cell surface plays a significant role in mediating EGF-induced EGFR clustering.

### 2.4. Evaluation of the Temperature-Dependent EGFR Clustering Induced by EGF Using N&B Analysis

With the successful development of EGFR endogenous protein expression tagged with GFP using CRISPR/Cas9, we further utilized EGFP-KI cells to investigate the effect of sialylation on the molecular clustering of EGFR-GFP by tracking the protein dynamics using RICS. Since EGFR signaling is involved in cluster formation [4,32], only limited information is available about the spatial and temporal dynamics of molecular clustering in live cells. RICS/N&B analysis allows us to investigate receptor clustering on the membrane in real-time live cell imaging on the basis of molecular diffusion and fluorescence intensity, as shown in Figure S4. To validate our cell model for N&B analysis, we assessed the molecular brightness of EGFR-GFP at varying temperatures (16 °C, 25 °C and 37 °C) to control the non-specific clustering of EGFR-GFP (Figure S5 in the SI). The results clearly showed that at lower temperatures (16 °C and 25 °C), EGF-induced internalization of EGFR-GFP was abolished even though the binding of EGF to EGFR was not affected at 25 °C (Figure S5B) and the corresponding brightness maps were uniform with a constant average brightness after EGF treatment (Figure S5B,C). In contrast, physiological temperature (37 °C) resulted in significant EGFR-GFP internalization and a more speckled brightness map with increased molecular brightness upon EGF stimulation (Figure S5A). Therefore, we chose 37 °C as the ideal temperature for analyzing the effect of sialidase on EGFR-GFP clustering using RICS and N&B analysis.

### 2.5. Residual Sialic Acid Mediates EGF-Induced EGFR Clustering

**Figure 6:**
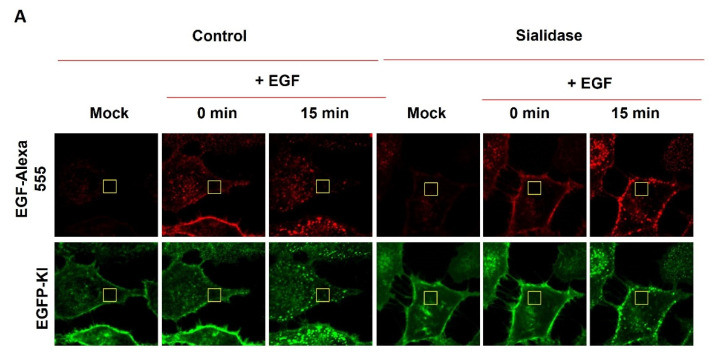

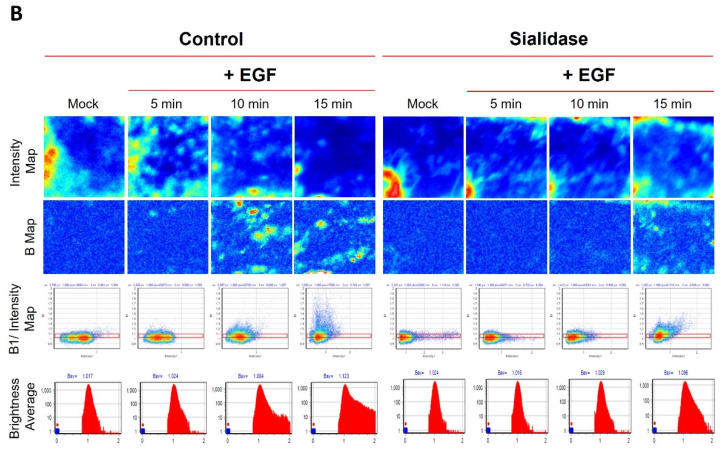
RICS measurement and N&B analysis of EGF-induced EGFR-GFP clustering in EGFP-KI cells treated with and without sialidase. (**A**) Confocal fluorescence images of EGFP-KI cells treated with and without sialidase prior to EGF stimulation. RICS images of the selected region of interest (ROI; yellow box) were acquired for N&B analysis; (**B**) N&B analysis of the ROI at intervals of 5 min after EGF treatment in the control and sialidase-treated EGFP-KI cells. The red boxes in the intensity maps represent monomeric EGFR-GFP defined by the mock control group for ease of comparison.

**Figure 7:**
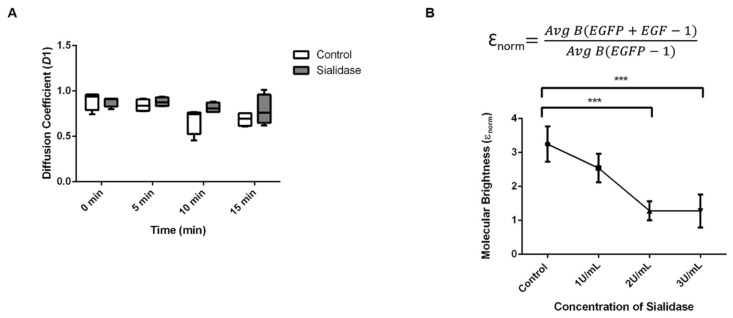
Molecular dynamics of EGFR-GFP clusters after EGF treatment. (**A**) Time-lapse analysis of the diffusion coefficient (D1) of EGFR-GFP clusters induced by EGF treatment in EGFP-KI cells pretreated with or without sialidase (N = 8 cells); (**B**) Molecular brightness (Ɛ_norm_) of cells treated with various concentrations of sialidase followed by EGF for 15 min. Avg B (EGFP + EGF) is the average brightness of EGFP induced by EGF, whereas Avg B (EGFP) is the average brightness of EGFP without EGF treatment. All data are presented as the means ± SEM of three independent experiments. *p* values were calculated using one-way ANOVA, with *** *p* ≤ 0.001 considered significant.

The role of sialylation in EGF-induced EGFR clustering was studied by RICS/N&B analysis in real-time live cell imaging. N&B is a well-established method to measure the number (N), brightness (B) and variance of the fluorescence intensity distribution of a fluorescent molecule [33]. In the N&B analysis, the molecular brightness is proportional to the variance of fluorescence fluctuation in the volume of observation. Monomeric GFP in EGFP-KI cells was used as a molecular brightness standard with an average brightness of 0.104 [34]. This quantitative information on molecular brightness can help to estimate the degree of oligomeric receptors and the dynamic change. Representative data obtained from the N&B analysis are presented in Figure 6. As expected, EGF-induced internalization of EGFR-GFP clusters was highly suppressed by sialidase treatment in EGFP-KI cells, as shown in the fluorescence images in Figure 6A. In control EGFP-KI cells, a more speckled brightness map was observed after EGF treatment for 10 min or longer. Meanwhile, the occurrence of a subpopulation with higher molecular brightness was apparent in both the intensity/brightness map and brightness histogram, indicating EGF-induced molecular clustering of EGFR-GFP (Figure 6B). Remarkably, in EGFP-KI cells pretreated with sialidase, EGF did not cause a substantial change in molecular brightness until 15 min, and the change was minor compared to those induced in control cells. Furthermore, the normalized molecular brightness, which is indicative of the degree of EGFR-GFP clusters after EGF treatment, was reduced by sialidase in a concentration-dependent manner (Figure 7B). Altogether, these results suggest that sialic acid residues are responsible for the formation of high degree EGFR-GFP clusters after EGF binding.

### 2.6. Diffusion Coefficient of EGFR-GFP in Desialylated EGFP-KI Cells

To confirm the formation of EGFR-GFP clusters, we sought to obtain information about the molecular diffusion of EGFR-GFP clusters after EGF treatment. In particular, RICS was employed to quantify the distribution of the lateral diffusion coefficient (D). As shown in Figure 7A, without EGF treatment (0 min), no significant change in the diffusion coefficient was observed upon sialidase treatment. In the control cells, the diffusion coefficient of EGFR-GFP was considerably lower after EGF treatment for 10 min or longer. This is in accordance with the change in the molecular brightness of EGFR-GFP from N&B analysis. Compared to the control group, which showed a slower diffusion rate of 0.688 ± 0.076 μm^2^/s after 15 min EGF treatment, the sialidase treatment group showed a faster diffusion rate of 0.789 ± 0.166 μm^2^/s. It could be the formation of a larger cluster with a restricted diffusion rate on the membrane. This is in agreement with our results from N&B analysis, which suggests the role of sialic acid residues in mediating EGF-induced EGFR-GFP clustering in EGFP-KI cells.

## 3. Discussion

Ligand-induced receptor clustering, which promotes spatial rearrangement of the receptor on the membrane for signaling [32], is an important cellular process implicated in certain pathophysiological conditions such as cancer [35] and pathogenic infection [36]. For studying receptor clustering, biochemical approaches such as chemical cross-linking and immunoprecipitation enable label-free measurement of protein clusters but lack adequate temporal resolution to resemble fast dynamic interactions in live cells. Fluorescence live-cell imaging can provide versatile means for studying receptor dynamics in real-time at an ultra-high sensitivity to the single-molecule level [37]. Protein overexpression is a powerful tool for fluorescently labeling proteins of interest for fluorescence imaging. However, the overexpressed receptor may alter the endogenous stoichiometry of receptor interactions, leading to ligand-independent interactions and activations [38]. Accordingly, by analyzing the consensus auto-phosphorylation at Tyr1068, our results indicate a significantly higher constitutive kinase activity (without EGF stimulation) of overexpressed EGFR-GFP in the transfected cells than in wild-type cells (Figure 1A). To address this issue, our strategy employed CRISPR/Cas9-mediated genome editing, through which we established EGFP-KI cells that expressed EGFR-GFP at close to the endogenous level. The EGFR-GFP in EGFP-KI cells resembles features of wild-type EGFR, including low constitutive kinase activity and EGF-dependent receptor activation, which allows for the study of receptor interactions in more physiological contexts. This CRISPR/Cas9-mediated fluorescent protein tagging is well-suited for studying protein dynamics at the normal expression level, complementary to the overexpression models, and can be adopted for smaller protein tags, such as HALO-, SNAP- and ACP-tag, for fluorescently labeling proteins of interest with smaller sizes

Additionally, in EGFP-KI cells, EGF induced a significant amount of intracellular EGFR-GFP clusters in a time-dependent manner (Figure 5), which is supported by the previous observations [39]. We used exogenous sialidase which can mimic the action of pathogenic sialidase during invasion, and demonstrated that the removal of sialic acid residuals of cell surface glycan by sialidase reduced EGF-induced EGFR-GFP clustering, which implicates the possible role of EGFR activation and repression during pathogen invasion. To support this observation, we employed RICS coupled with N&B analysis to study the molecular dynamics of EGFR-GFP clusters induced by EGF due to their high sensitivity [40]. We characterized the diffusion coefficient and molecular brightness of EGFR-GFP in live EGFP-KI cells and confirmed that sialic acid residues are essential for the formation of EGFR-GFP clusters (Figure 6 and Figure 7). Overall, our results reveal that the EGFR-GFP cluster during EGF-induced internalization and sialic acid residues play a role in mediating this process. This finding complements the previous studies that demonstrated the role of sialylation in EGFR activation and interactions using different approaches and experimental settings [16,17,18,19,20]. Not included in the present study are the mechanistic studies on how sialylation controls the clustering of EGFRs. It has been shown that sialidase-mediated desialylation contributes to conformational changes and affects the function of glycoproteins [41]. Therefore, sialoside-terminated glycans bearing negative charges on EGFR might influence the recognition of EGF ligands and hence the dimerization of EGFRs [42]. According to our observations, it is believed that sialic acid residues could mediate the interactions between EGFRs to cluster rather than affect EGF binding to EGFR. Furthermore, since the action of sialidase is global, it should not rule out the possibility that sialic acids on other glycoconjugates such as gangliosides of lipid rafts could mediate the interplay with EGFRs, leading to the formation of EGFR clusters.

From another perspective, EGFRs are one of the major sialylated proteins in regulating gene expression profiles attributed to cancer cell pathogenicity [43]. Overexpression of β-galactoside α-2,6-sialyltransferase, which increases α-2,6 sialylation of EGFR, has been linked to poor prognosis of cancers [29] and has been implicated in promoting tumor cell proliferation and drug resistance in some cancers [16,17]. Our observed results may also provide new insight into how sialylation affects EGFR dynamics and its role in cancer progression. CRISPR/Cas9-mediated fluorescence tagging can provide a versatile means in conjunction with imaging techniques for unbiasedly studying protein dynamics, including but not limited to EGFRs. The established paradigm can be expanded to reveal EGFR behaviors under other disease conditions, such as autoimmune [44], neurodegenerative [45], and cardiovascular diseases [46].

## 4. Materials and Methods

### 4.1. Chemicals and Materials

Dulbecco’s modified Eagle’s medium (DMEM), fetal bovine serum (FBS), sodium pyruvate, penicillin streptomycin, reduced serum media (Opti-MEM), Dulbecco’s phosphate-buffered saline (PBS), Lipofectamine 2000, Hoechst 33342, Alexa Fluor^TM^ 555 EGF complex (EGF-Alexa Fluor 555), EGF recombinant protein, BCA protein assay kit (BCA) and ProLong diamond antifade mounting medium (ProLong^TM^ Diamond) and Protein G agarose were purchased from Invitrogen (Carlsbad, CA, USA). Neuraminidase (sialidase) from *Clostridium perfringens* and dibenzocyclooctyne-PEG_4_-Fluor 545 (Fluor 545) were purchased from Sigma-Aldrich (St. Louis, MO, USA). The other chemicals and reagents used were purchased from different suppliers, including NP-40 (Calbiochem, San Diego, CA, USA), protease inhibitor cocktail set II EDTA-free (Merck Millipore, Darmstadt, Germany), paraformaldehyde (Electron Microscopy Sciences, Fort Washington, PA, USA), 30% acrylamide/bis-acrylamide (Bio-Rad Laboratories Ltd., Mississauga, ON, Canada) and ECL Western blotting substrate (PerkinElmer, Boston, MA, USA). Peracetylated azido-*N*-acetylmannosamine (Ac_4_ManNAz) and peracetylated *N*-acetylmannosamine (Ac_4_ManNAc) were synthesized.

### 4.2. Antibodies and Plasmids

Rabbit anti-EGFR antibodies (D38B1) and phosphor-EGF receptor Tyr1068 (2236) were purchased from Cell Signaling Technology (Danvers, MA, USA). The mouse anti-β-actin antibody (A5441) was purchased from Sigma-Aldrich (Oakville, ON, Canada). The mouse anti-GFP antibody (JL-8) was obtained from Clontech (Palo Alto, CA, USA). Anti-EGFR mAb (cetuximab C225) was purchased from MedChemExpress (Shanghai, China). Horseradish peroxidase (HRP)-conjugated goat anti-mouse and anti-rabbit antibodies were purchased from Jackson Immuno-research Inc. (West Grove, PA, USA). The EGFR-GFP transfection plasmid (32,751) was purchased from Addgene (Watertown, MA, USA), and a DNA ligation kit (6022) was obtained from Takara Bio Inc. (Shiga, Japan).

### 4.3. Cell Cultures

Cells were maintained at 70–80% confluence and cultured in DMEM supplemented with 10% FBS together with sodium pyruvate and penicillin/streptomycin. They were cultured in a humidified chamber with 5% CO_2_ at 37 °C.

### 4.4. Constructs and sgRNA/Cas9 Plasmid

pAll-Cas9. The Ppuro plasmid was obtained from C6 Core Lab, Academia; it contains two BsmBI restriction endonuclease sites upstream of the sgRNA scaffold and the guide sequences of AGGGCTCATACTATCCTCCG (without the protospacer adjacent motif (PAM) sequence). Both guide sequences were generated with oligonucleotide modifications to insert BsmBI restriction sites. BsmBI was used to digest pAll-Cas9. The Ppuro backbone and the abovementioned double-strand guide sequences were annealed using a DNA ligation kit. The final bicistronic vector encoded the gRNA and the CAS9 nuclease. All plasmids used in this study were validated with dideoxyribonucleotide sequencing by Genomics (New Taipei City, Taiwan).

### 4.5. Construction of EGFP-KI Donor Vectors

The homology arms of the donor vector were synthesized (Integrated DNA Technologies, Coralville, IA, USA) to design the donor template DNA for homology-directed repair (HDR). The donor vector comprised a 359 bp left homology arm (LHA), GFP fragment and 279 bp right homology arm (RHA) in the pUC19 backbone.

### 4.6. Establishment of the EGFP-KI Cells

HeLa cells were plated on 60-mm culture plates and transfected with 1 μg of pAll-Cas9-sgRNA vectors and 1 μg of KI donor DNA using Lipofectamine 2000, followed by puromycin selection. Three days after transfection, transfected cells were seeded onto 100-mm culture plates. Selected cells were analyzed and sorted using a BD FACSJazz^TM^ Cell Sorter (BD Biosciences, San Jose, CA, USA) to isolate EGFP-KI cells. GFP-positive cells were subjected to limiting dilution for single-cell cloning.

### 4.7. Validation of EGFP-KI Cells Using Immunoblotting, Flow Cytometry and Confocal Microscopy

EGFP-KI cells were assessed for phosphorylation and EGFR expression using Western blotting. HeLa cells lacking GFP expression served as a control. The membrane was blocked for 1 h at room temperature and probed with anti-EGFR (1:2000) and anti-β-actin (1:2000) antibodies, which served as an internal control overnight at 4 °C. The membranes were washed with 5% TBST 5 times and subsequently incubated with HRP-conjugated goat anti-rabbit and anti-mouse antibodies. Finally, proteins were visualized using ECL blotting substrates with an iBright^TM^ FL1000 Imaging System (Thermo Fisher Scientific, Inc., Waltham, MA, USA).

For flow cytometry, cells were grown on 60-mm culture plates, detached with EDTA and resuspended in PBS containing 1% FBS at 4 °C. Fluorescence intensity was analyzed using fluorescence-activated cell sorting with a FACSCalibur^TM^ flow cytometer (BD Biosciences, San Jose, CA, USA). In the confocal imaging study, prewashed EGFP-KI cells were incubated with 100 ng/mL EGF-Alexa Fluor 555 at 5-min intervals (5, 10, and 15 min). Cells were fixed, and nuclei were counterstained with Hoechst and mounted with ProLong^TM^ Diamond overnight. Fluorescence images were captured using an LSM 980 confocal microscope (Zeiss, Oberkochen, Germany) with excitation wavelengths of 405 nm for Hoechst, 488 nm for EGFP-KI and 563 nm for EGF-Alexa Fluor 555. The fluorescence of the cells was detected at 410–470 nm, 490–552 nm, and 567–760 nm, respectively.

EGFP-KI cells were treated with 3 U/mL sialidase for 1 h at 37 °C to analyze the fluorescent signal in confocal images and flow cytometry. EGFP-KI cells were lysed, and proteins were immunoblotted with antibodies against EGFR (1:2000), EGFP (1:4000) and the internal control β-actin (1:2000) overnight at 4 °C. The membranes were incubated with HRP-conjugated goat anti-rabbit and anti-mouse antibodies diluted 1:20,000. Finally, proteins were visualized using ECL blotting substrates and an iBright^TM^ FL1000 Imaging System (Thermo Fisher Scientific, Inc., Waltham, MA, USA).

### 4.8. EGFR-GFP-Expressing Cells (Transfection and CRISPR/Cas9), Western Blotting and Fluorescence Confocal Microscopy Imaging

HeLa cells were seeded at 70–80% confluence and incubated overnight in 60-mm culture plates, and the EGFR-GFP plasmid was transfected with a transfection reagent (Lipofectamine 2000) according to the manufacturer’s protocol. HeLa cells and CRISPR/Cas9-derived EGFP-KI cells were treated with recombinant EGF protein for 15 min at 37 °C. HeLa cells, EGFP-KI and transfected EGFR-GFP were lysed with lysis buffer containing 100 mM sodium phosphate (pH 7.4), 150 mM NaCl, 10% glycerol, 1% NP-40 and 1× protease inhibitor cocktail set II EDTA free. After lysis on ice for 30 min, lysates were centrifuged at 13,000 rpm for 15 min at 4 °C. Supernatants were collected, and protein concentrations were measured using a BCA protein assay kit. Lysates were separated on acrylamide/bis-acrylamide SDS–PAGE gels and then transferred onto Protran^TM^ nitrocellulose membranes (Whatman, Dassel, Germany) with antibodies against phospho-Y 1068 and EGFR with β-actin as an internal control. The membranes were washed with 5% Tween-20 Tris-buffered saline (TBST) 5 times and subsequently incubated with HRP-conjugated goat anti-rabbit and anti-mouse (1:20,000) antibodies. Finally, proteins were visualized after incubation with ECL blotting substrates using electrochemiluminescence with an iBright^TM^ FL1000 Imaging System (Thermo Fisher Scientific, Inc., Waltham, MA, USA).

For confocal imaging, cells were seeded at 70–80% confluence on coverslips in 12-well plates and incubated overnight. Cells were incubated with 100 ng/mL EGF-Alexa Fluor 555 for 15 min at 37 °C. Cells were fixed with 3% paraformaldehyde, nuclei were counterstained with Hoechst, and coverslips were mounted with ProLong^TM^ Diamond overnight. Fluorescence images were captured with an LSM 980 confocal microscope (Zeiss, Oberkochen, Germany) using a 63×/1.4 objective and excitation wavelengths of 405 nm for Hoechst, 488 nm for EGFR-GFP and 563 nm for EGF-Alexa Fluor 555. The fluorescence of the cells was detected at 410–470 nm, 490–552 nm, and 567–760 nm, respectively.

### 4.9. Determination of Sialic Acid Cell Surface Labeling

EGFP-KI cells were seeded at 70–80% confluence on coverslips in 12-well plates and cultured overnight. They were metabolically labeled with 100 µM Ac_4_ManNAz, and Ac_4_ManNAc served as a negative control and were incubated at 37 °C for 3 days. Prior to labeling, cells in all wells were washed with warmed Opti-MEM. Then, the cells were labeled with 25 µM Fluor 545 at 4 °C for 30 min and washed with 1% ice-cold FBS in Opti-MEM 4 times. For sialidase treatment, cells were pretreated with 3 U/mL sialidase for 1 h at 37 °C before Fluor 545 labeling. Cells were fixed, nuclei were counterstained with Hoechst, and coverslips were mounted with ProLong^TM^ Diamond overnight. Fluorescence images were captured with an LSM 980 confocal microscope (Zeiss, Oberkochen, Germany) at excitation wavelengths of 405 nm for Hoechst, 488 nm for EGFP-KI and 558 nm for Fluor 545 lasers. The fluorescence of cells was detected between 410–470 nm, 490–552 nm and 561–632 nm, respectively.

Cells were pretreated with sialidase and labeled with Fluor 545 in 60 mm culture plates for immunoprecipitation, and cells were lysed with lysis buffer. Lysates were incubated with 2 µg of anti-EGFR mAb for 2 h at 4 °C with agitation. Protein G agarose beads (50% *v*/*v*) were added and agitated overnight at 4 °C. Immunoprecipitates were washed three times with 1 mL of lysis buffer at 2500 rpm for 5 min. Fluor 545-labeled protein lysates were dissolved in SDS sample buffer containing 10% 2-mercaptoethanol and heated at 95 °C for elution. SDS–PAGE was performed using a Typhoon imager (Amersham Bioscience, Amersham, UK) to detect Fluor 545 signals. For subsequent Western blotting, samples were resolved on SDS–PAGE gels and transferred to nitrocellulose membranes. The membrane was probed with an anti-EGFR antibody (1:2000) followed by an HRP-conjugated goat anti-rabbit antibody (1:20,000), and the membrane was visualized using the electrochemiluminescence of ECL blotting substrates with an iBright^TM^ FL1000 Imaging System (Thermo Fisher Scientific, Inc., Waltham, MA, USA).

### 4.10. EGF Ligand-Induced Kinetic Studies after Treatment with Sialidase

EGFP-KI cells were grown on cover glasses placed in 12-well plates at 70–80% confluence. Cells were washed with Opti-MEM once and treated with 3 U/mL sialidase for 1 h at 37 °C. Prewashed cells were incubated with 100 ng/mL EGF-Alexa Fluor 555 at 5 min intervals (5, 10, 15 and 20 min) at 37 °C. Cells were incubated with different concentrations of sialidase (1 U/mL, 2 U/mL and 3 U/mL) for 1 h at 37 °C, followed by 100 ng/mL EGF-Alexa Fluor 555 for 15 min at 37 °C. Cells were fixed, nuclei were counterstained with Hoechst and coverslips were mounted with ProLong^TM^ Diamond overnight. Fluorescence images were captured with an LSM 980 confocal microscope (Zeiss, Oberkochen, Germany) with excitation wavelengths of 405 nm for Hoechst, 488 nm for EGFP-KI and 563 nm for EGF-Alexa Fluor 555. The fluorescence of cells was detected between 410–470 nm, 490–552 nm and 567–760 nm, respectively.

### 4.11. High-Content Analysis of the Count, Area, and Intensity of EGFP-KI Cells

The high content analysis was performed using a benchtop CQ1 high content analyzer (Yokogawa Electric Corp., Tokyo, Japan) for high-throughput screening. Images were segmented based on the contours of the nucleus and punctate structures of the internalized fluorescent signal for EGF-Alexa Fluor 555 (Figure S1). Data were collected and analyzed by determining the counts, area and intensity of each sample with approximately equal average cell numbers (N = 1000).

### 4.12. Calibration and Instrumentation Setup

Water immersion objective lenses with a high numerical aperture (NA) were corrected with motor alignment. Calibration was performed by measuring the autocorrelation function *G*(t) with rhodamine 110 in PBS, which has a diffusion coefficient (*D*) of 420 ± 30 µm^2^/s. The radial waist acquired was 0.36 ± 0.05 µm. The condition was optimized by measuring counts per second (CPS) and photon counts. The process was repeated several times with the reference standards, and fitting curves were established using 3D Gaussian.

### 4.13. Sample Preparation for RICS Imaging

Before imaging, EGFP-KI cells were seeded in an 8-well glass chamber slide Nunc^®^ Lab-Tek^®^ II (Nunc Inc., Neperville, IL, USA) at 70–80% confluence. Cells were pretreated with 3 U/mL sialidase for 1 h at 37 °C. Prewashed cells were incubated with 100 ng/mL EGF-Alexa Fluor 555, and a series of RICS images were captured at 5 min intervals at 37 °C. Different concentrations of sialidase (1 U/mL, 2 U/mL, and 3 U/mL) were incubated with the cells for 1 h at 37 °C, followed by 100 ng/mL EGF-Alexa Fluor 555 for 15 min at 37 °C. For the analysis of the temperature-dependent measurement, the temperatures were adjusted to 16 °C, 25 °C and 37 °C in a temperature-controlled chamber.

### 4.14. Data and Acquisition of RICS Images

An Eclipse Ti-U inverted microscope with a submicrometer automatic controlled *XYZ* stage (Nikon, Tokyo, Japan) in photocount mode was used for the RICS measurement using a 60×, 1.42 NA water immersion objective. The operating system was equipped with a pulsed laser that was controlled by ISS VistaVision software Version 4.2.193.0 (Champaign, IL, USA). The fluorescence signal for the green emission channel had a nominal bandwidth of a 525/540 nm bandpass filter (Chroma Technology Corp., Rockingham, VT, USA). EGF-Alexa Fluor 555 was passed through a 600/637 nm bandpass filter (Chroma Technology Corp., Rockingham, VT, USA).

A time series of 100 frames of 256 × 256 pixels with a size of 0.05 µm/pixel and a scan speed of 20 µs/pixel was used (Figure S4). According to the previous study, the pixel dwell time should be maintained between 8–20 μs because a prolonged dwell time may lead to photobleaching [47]. RICS measurements were performed at the apical level of the membrane to avoid interference with the glass surface. For time-lapse imaging of live cells following EGF treatment, images were acquired at 0 min, 5 min, 10 min and 15 min. The pinhole size was adjusted to 100 nm, and the acquisition laser power was 50% for 488 nm and 60% for 561 nm. The diffusion coefficient was obtained using the RICS function after subtracting the moving average background (N = 10 frames).

### 4.15. Analysis of N&B Measurements

All the N&B data were processed and analyzed using the SimFCS 4 program developed by Laboratory Fluorescence Dynamics by Enrico. Raw data were analyzed and fitted to the intensity profile with 2D diffusion fitting to all surfaces. All 100 frames captured at different time points were subjected to a moving average of 10 frames. The slow fluctuation attributed to cellular movements was removed through this process.

### 4.16. Statistical Analysis

Data were collected from more than 3 independent experiments and were analyzed using GraphPad Prism 5.0 software (GraphPad Software Inc., San Diego, CA, USA). Statistically significant differences were examined using one-way and two-way ANOVA to derive the significance of the differences between two groups. *p* < 0.05 was considered significant.

## 5. Conclusions

We established a CRISPR/Cas9 EGFP-KI cell model that expresses GFP-tagged EGFRs at endogenous levels. In live EGFP-KI cells, sialidase decreased the sialic acid content on EGFR and resulted in a reduction in the EGF-induced internalization of EGFR-GFP clusters under confocal fluorescence microscopy. RICS/N&B analysis further demonstrated a significant increase in EGFR-GFP cluster formation after EGF treatment in the absence of sialidase. Overall, our results reveal that sialic acid residues play a role in mediating EGFR-GFP clustering during EGF-induced internalization. This study provides new insight into the use of sialidases to remodel host glycoproteins during viral and bacterial infections. On the other hand, the CRISPR/Cas9-mediated fluorescent tagging of endogenous proteins in combination with fluorescence imaging techniques can be a versatile toolkit for studying the molecular dynamics and interactions of proteins under physiological and pathological conditions.

## Data Availability

All data in this study are presented in this manuscript and the Supplementary Information.

## Supplementary Materials

The supporting information can be downloaded at: https://www.mdpi.com/article/10.3390/ijms23158754/s1.
