# Supplementary material for: A Multiscale Molecular Dynamic Analysis Reveals the Effect of Sialylation on EGFR Clustering in a CRISPR/Cas9-Derived Model
Source: Int J Mol Sci. 2022 Aug 6;23(15):8754. doi: 10.3390/ijms23158754 (PMC9368999; doi:10.3390/ijms23158754)
Supplement: Supplementary file 1 [file ijms-23-08754-s001.zip › ijms-1835353-supplementary.pdf]

Supplementary Information

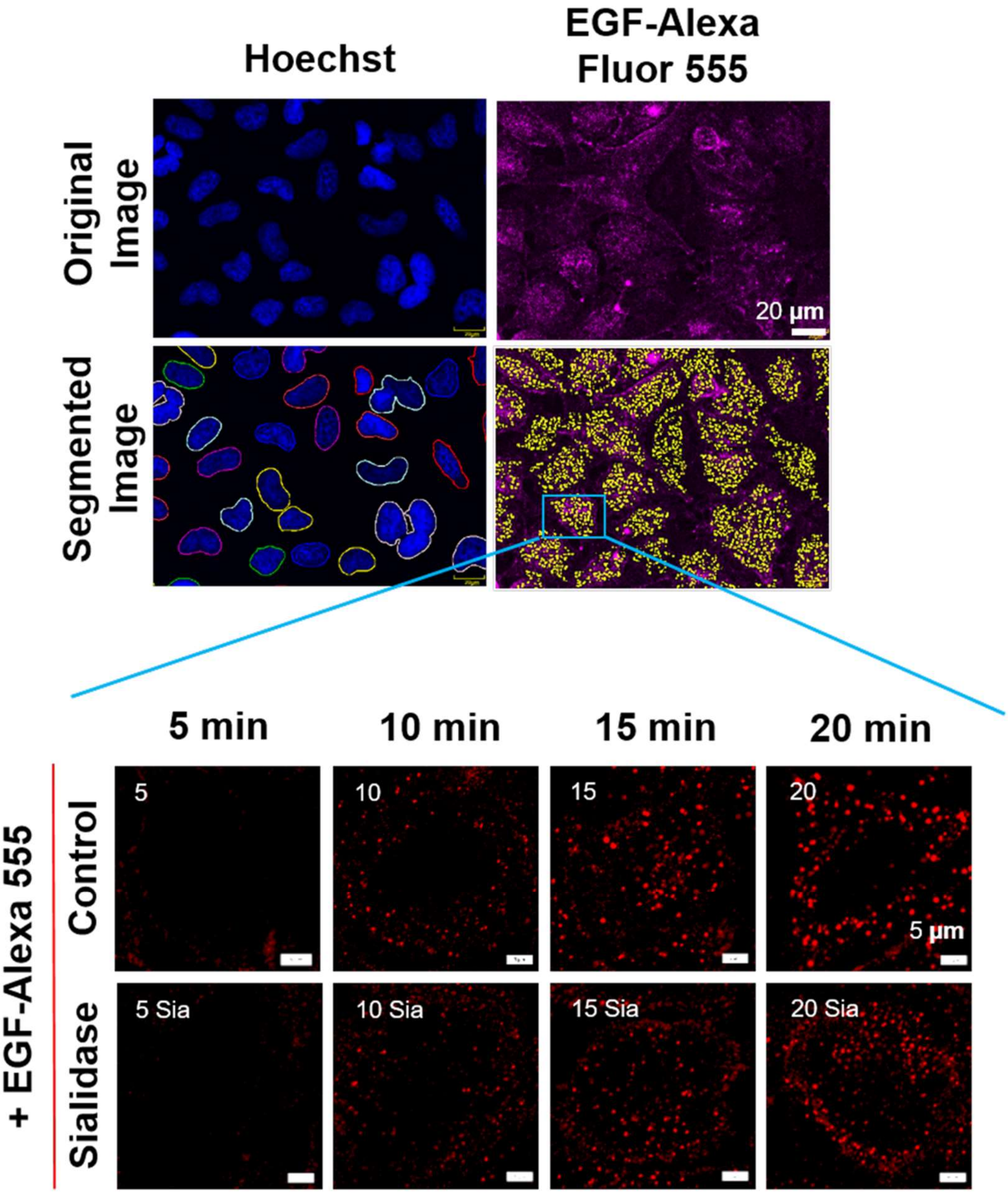

Figure S1. Segmentation of the high content analysis map of Hoechst and EGF-Alexa Fluor 555 in the control and sialidase treatment groups stimulated with EGF for 5 to 20 min at 37 °C.

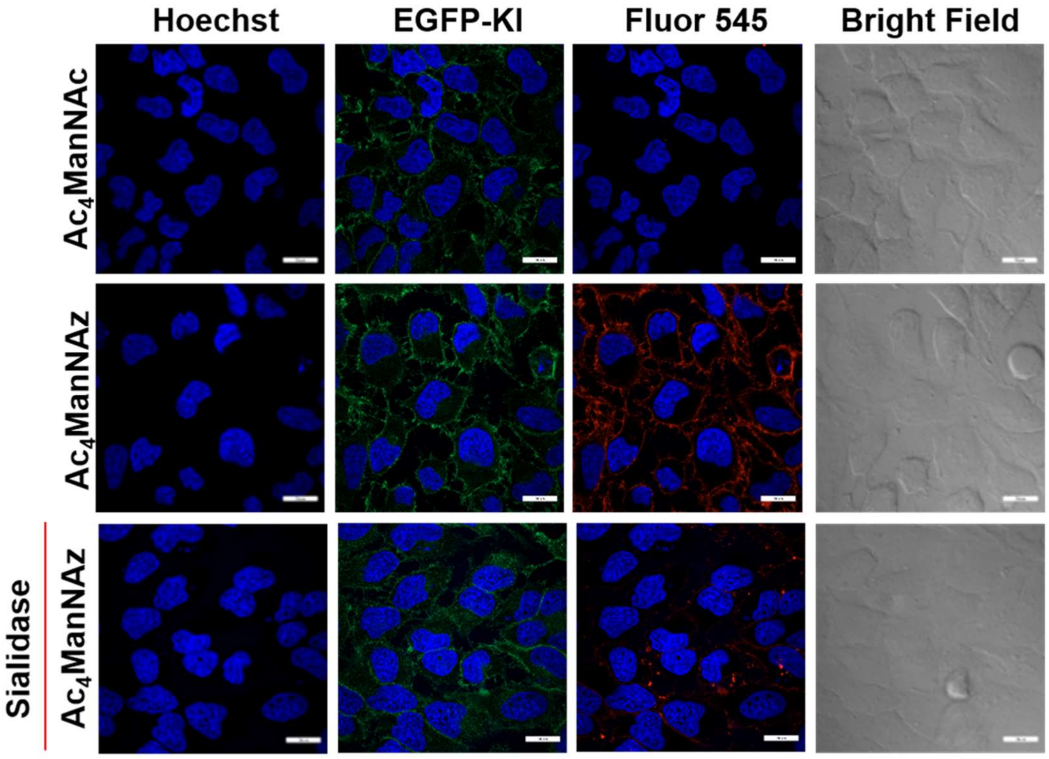

Figure S2. Fluorescence images of sialylated EGFP-KI cells by sialidase treatment. Cells were treated with $Ac_4ManNAz$, and 3 U/mL sialidase was added prior to SiaNAz labeling with Fluor 545.

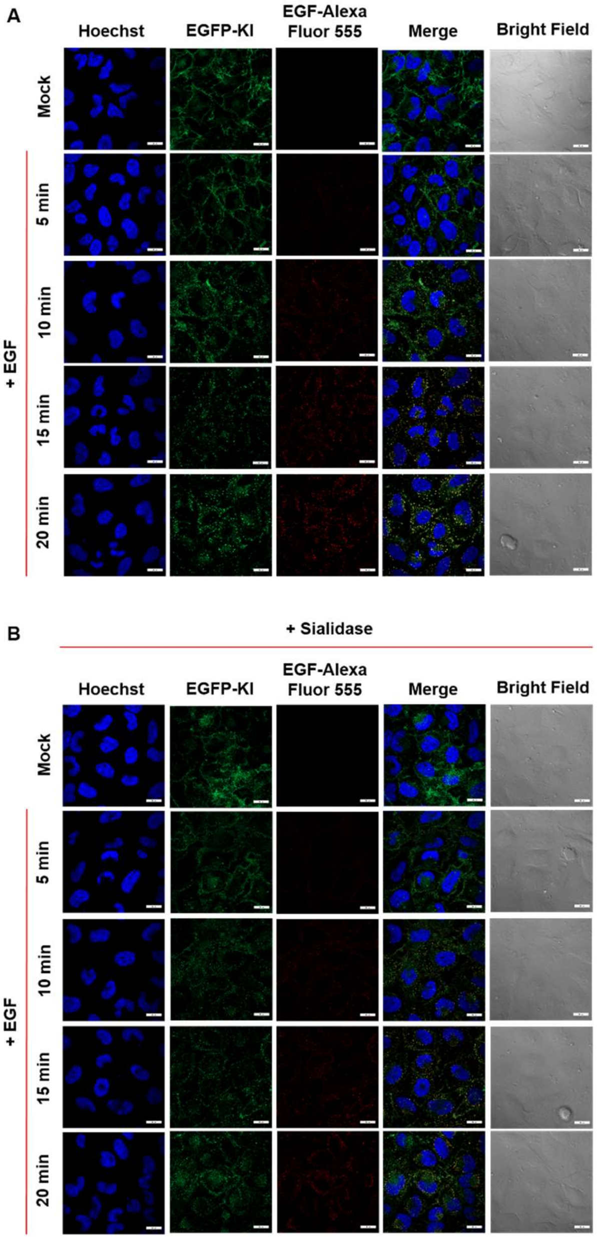

Figure S3. Time-lapse images of desialylated ligand-induced (EGF) EGFP-KI glycoconjugates. (**A**) EGFP-KI cells were treated with 100 ng/mL EGF-Alexa Fluor 555 for 5–20 min at 37 °C. (**B**) EGFP-KI cells were incubated with sialidase (3 U/mL sialidase) to catalyze sialic acid removal, followed by 100 ng/mL EGF-Alexa Fluor 555 for 5–20 min at 37 °C. Scale bar, 20 μm.

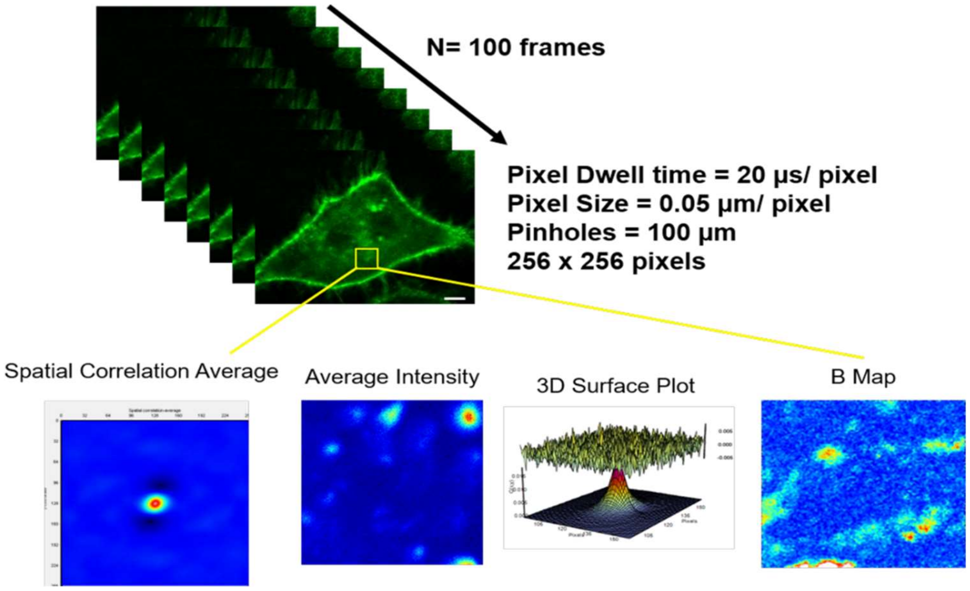

Figure S4. Schematic representation of the principle of raster scanning imaging of EGFP-KI cells using consecutive acquisitions for 100 frames. Scale bar, 10 μm.

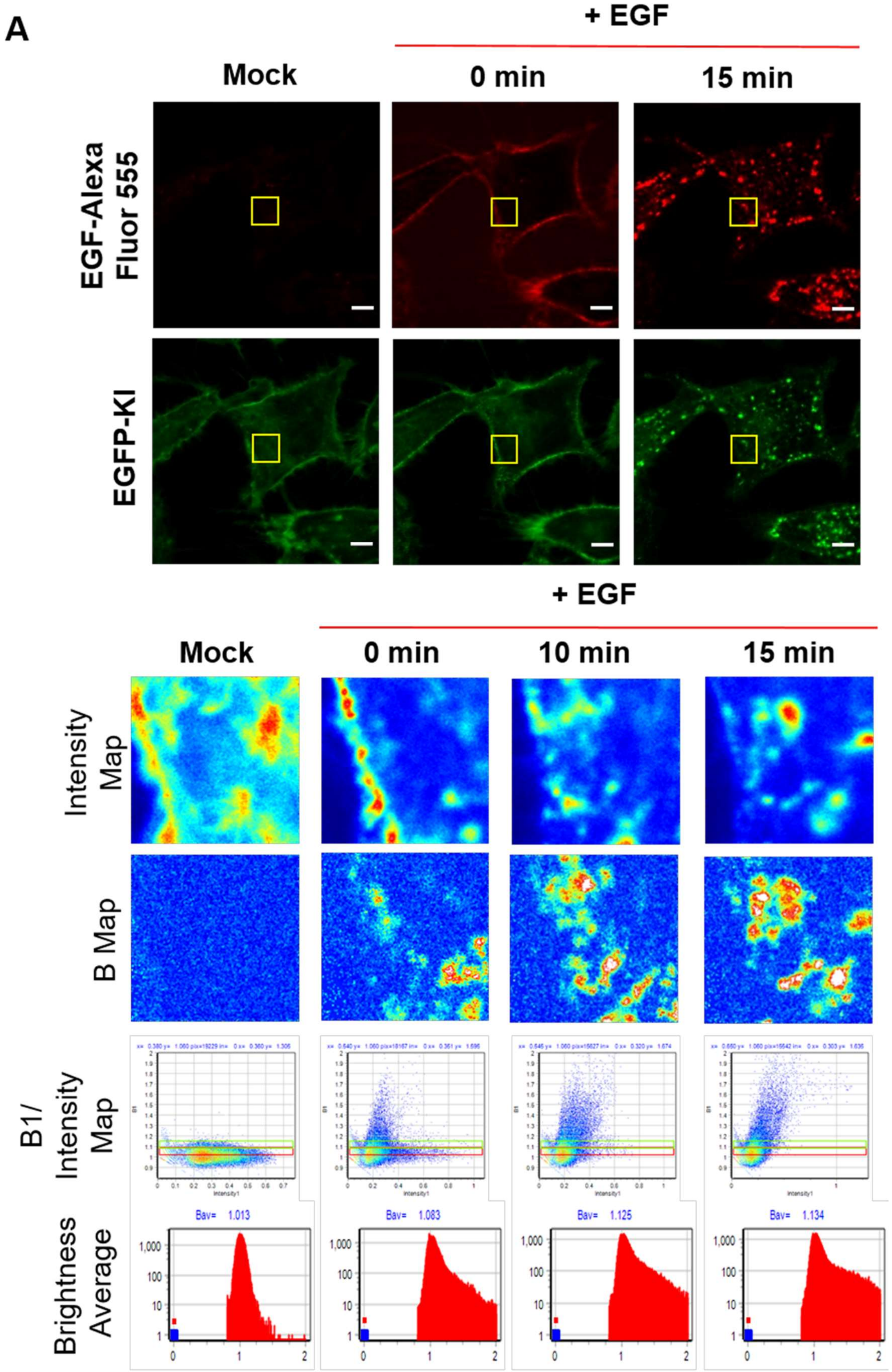
A
+ EGF
Mock
0 min
15 min
EGF-Alexa Fluor 555
EGFP-KI
+ EGF
Mock
0 min
10 min
15 min
Intensity Map
B Map
B1/ Intensity Map
Brightness Average
Bav= 1.013
Bav= 1.083
Bav= 1.125
Bav= 1.134

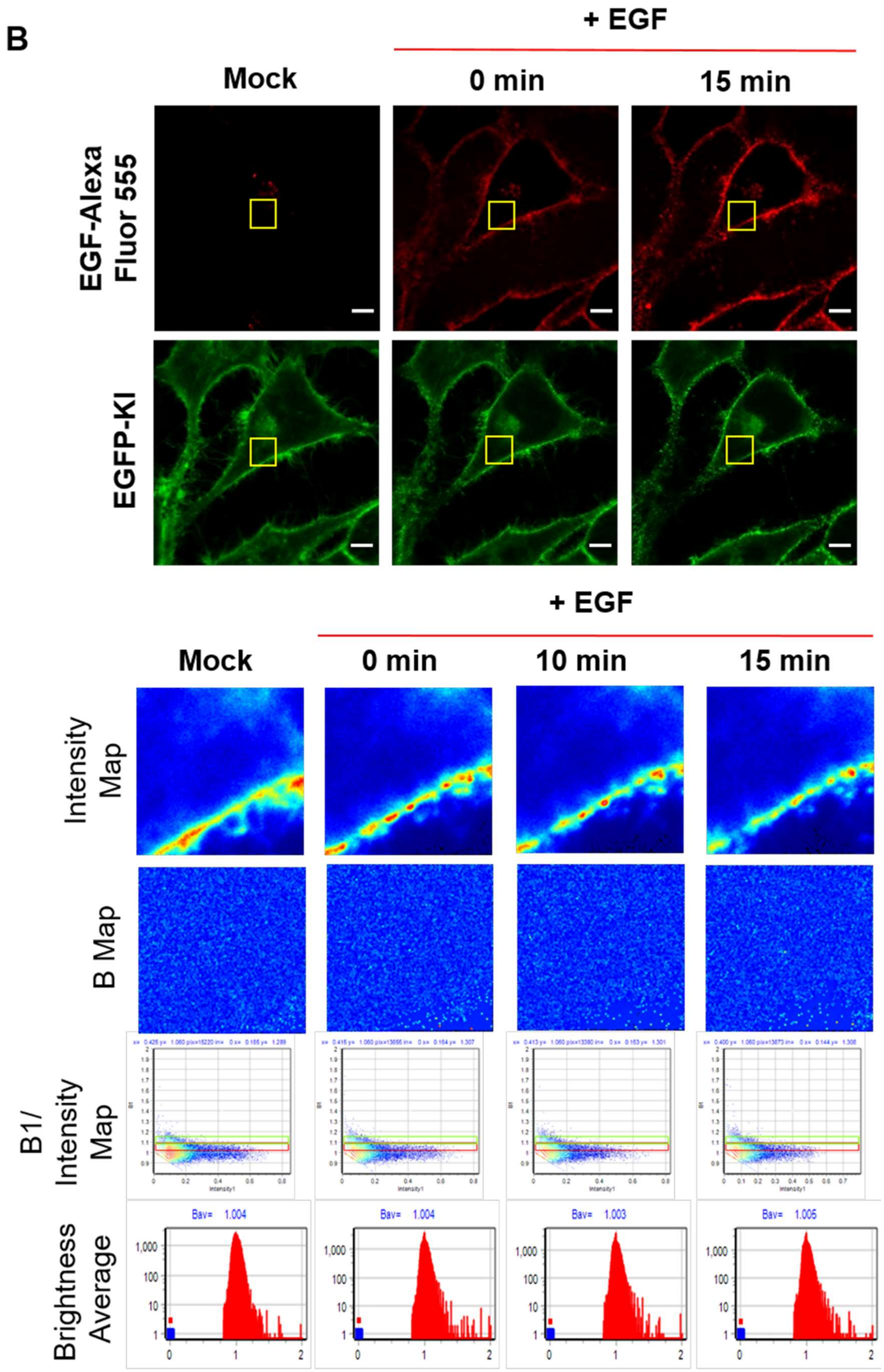
B
+ EGF
Mock
0 min
15 min
EGF-Alexa Fluor 555
EGFP-KI
+ EGF
Mock
0 min
10 min
15 min
Intensity Map
B Map
B1/ Intensity Map
Brightness Average
Bav= 1.004
Bav= 1.004
Bav= 1.003
Bav= 1.005

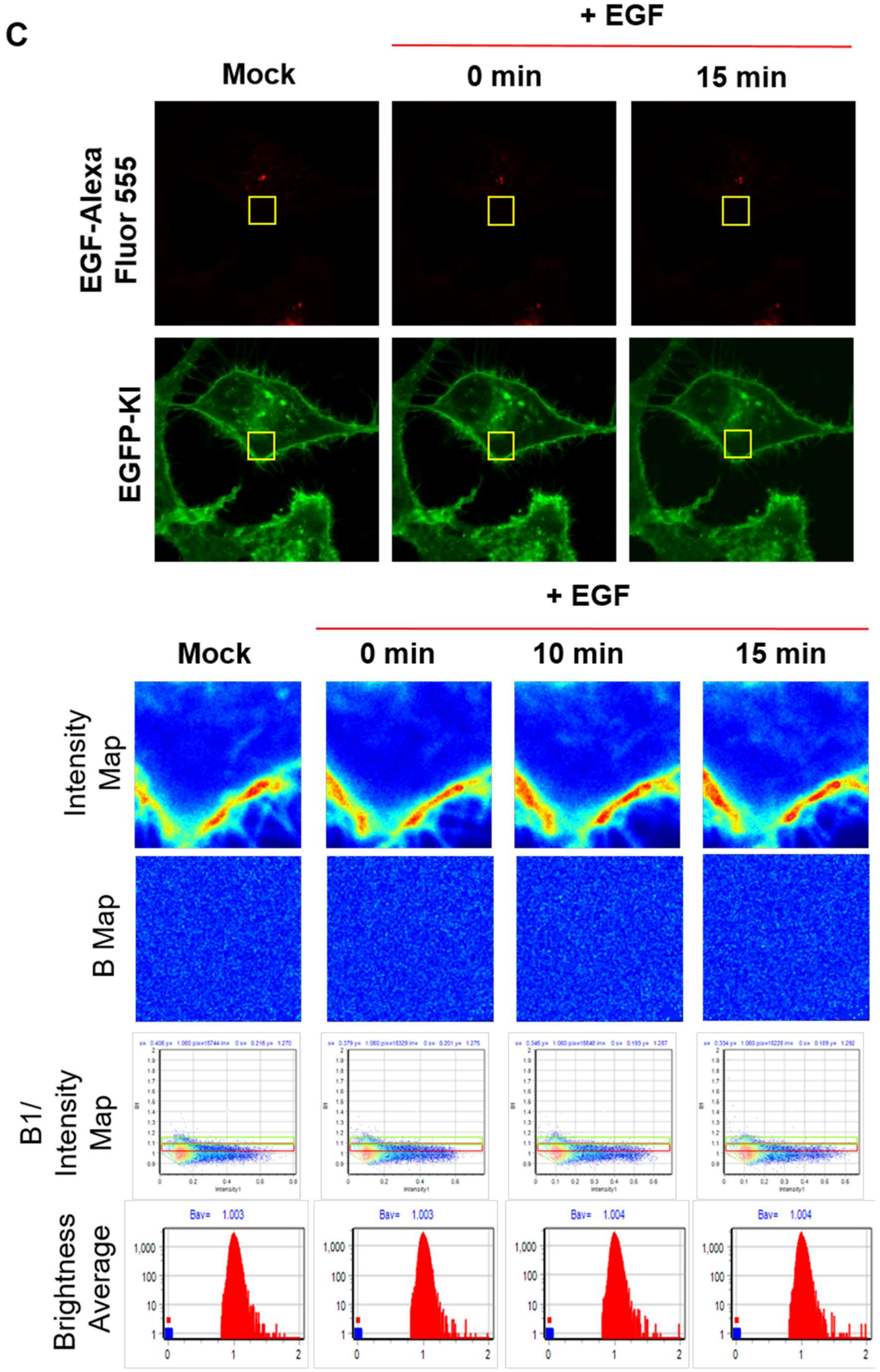
C
+ EGF
Mock
0 min
15 min
EGF-Alexa Fluor 555
EGFP-KI
+ EGF
Mock
0 min
10 min
15 min
Intensity Map
B Map
B1/ Intensity Map
Brightness Average
Bav= 1.003
Bav= 1.003
Bav= 1.004
Bav= 1.004

Figure S5. N&B analysis of EGFR-GFP in EGFP-KI cells maintained at different temperatures and treated with 100 ng/mL EGF for 0 min, 10 min and 15 min: (**A**) 37 °C, (**B**) 25 °C and (**C**) 16 °C.
